# Precision and Efficacy of Digital Technology in Orthognathic Surgery for Facial Asymmetry Correction: A Quantitative Analysis

**DOI:** 10.1007/s00266-025-04974-x

**Published:** 2025-06-13

**Authors:** Xiang Sun, Jiusong Han, Jing Wang, Junxiang Lian, Jingpeng Liu, Simin Li, Shuguang Liu, Huixi Zhou

**Affiliations:** Department of Oral and Maxillofacial Surgery, Stomatological Hospital, School of Stomatology, Southern Medical University, 366 South of Jiangnan Boulevard, Haizhu District, Guangzhou, 510280 Guangdong China

**Keywords:** Digital technology, Facial asymmetry, Orthognathic surgery, Symmetry

## Abstract

**Objectives:**

To evaluate the efficacy of digital technology-assisted design in orthognathic surgery for patients with facial asymmetry.

**Materials and Methods:**

The study comprised 30 patients with facial asymmetry. Mimics software was used to digitally simulate surgical plans and create guide plates for orthognathic surgery. The midsagittal plane, Frankfurt horizontal plane, and coronal plane of the perosseous nasion point served as reference planes to evaluate three-dimensional consistency between preoperative simulation designs and postoperative three-dimensional cranial models, specifically examining bilateral maxillary and mandibular canine cusps and mesiobuccal cusps of first molars. Distance differences between bilateral corresponding landmarks on postoperative models and the three reference planes were measured, asymmetry rates calculated, and the distance from pogonion to midsagittal plane measured to evaluate facial hard tissue symmetry after surgery.

**Results:**

No significant differences were observed between preoperative simulation designs and postoperative three-dimensional cranial models regarding the distances between bilateral corresponding landmarks and the three reference planes. Postoperatively, distance differences between bilateral corresponding landmarks and the three reference planes were ≤ 1 mm. Seventy-five percent of asymmetry rate indicators were less than 10%. The mean distance from pogonion to midsagittal plane was 1.82 ± 1.16 mm.

**Conclusion:**

The application of digital technology in orthognathic surgery for patients with facial asymmetry enhances operative precision and ensures favorable treatment outcomes.

**Clinical Relevance:**

Digital technology in orthognathic surgical planning improves treatment effectiveness for patients with facial asymmetry.

**Level of Evidence III:**

This journal requires that authors assign a level of evidence to each article. For a full description of these Evidence-Based Medicine ratings, please refer to the Table of Contents or the online Instructions to Authors www.springer.com/00266.

## Introduction

Facial asymmetry represents a common dentofacial deformity that presents significant therapeutic challenges. The reported prevalence ranges from 21 to 85%, with variations attributed to study population characteristics, assessment methodologies, and symmetry criteria employed [1]. Clinical manifestations typically include bilateral facial asymmetry, unilateral chin deviation, occlusal plane inclination, dental midline discrepancies in both arches, and malocclusion. Facial symmetry serves as a critical aesthetic parameter with substantial implications for a patient's physical appearance, masticatory function, speech articulation, and psychological well-being [2]. Orthognathic surgery constitutes an essential approach for addressing facial asymmetry deformities, involving strategic osteotomy and repositioning of the maxillofacial skeleton to correct facial irregularities and restore proper dental occlusion [3]. Given the complex three-dimensional anatomy of the maxillofacial region, meticulous preoperative planning is indispensable. Asymmetric facial deformities frequently involve three-dimensional malpositions of the maxilla and mandible, requiring precise rotational and translational movements to achieve facial harmony. Traditional planning modalities—including two-dimensional cephalometric analysis, conventional surgical predictions, and model surgery—fail to adequately simulate these complex three-dimensional relationships, often resulting in suboptimal clinical outcomes [4].

Recent advancements in cone-beam computed tomography (CBCT) and digital technology have revolutionized this field, enabling the creation of detailed three-dimensional cranial reconstructions. This digital framework facilitates comprehensive preoperative assessment, surgical simulation, outcome prediction, and postoperative evaluation. The implementation of digitally designed osteotomy guides and occlusal splints ensures accurate translation of virtual surgical plans to the operating theater, thereby enhancing correction precision for facial asymmetry deformities [5–7]. Despite the increasing adoption of digital planning techniques in orthognathic surgery, there remains a significant research gap regarding standardized quantitative evaluation of surgical outcomes specifically for facial asymmetry correction. Previous studies have focused primarily on technical feasibility or general surgical accuracy, but few have established comprehensive measurement protocols with defined thresholds for successful symmetry restoration. Additionally, while the superiority of digital approaches over traditional methods has been suggested, robust comparative data with consistent evaluation parameters remains limited, particularly for the challenging subset of patients with pronounced facial asymmetry.

To address these knowledge gaps and validate the efficacy of digital technology in the surgical management of facial asymmetry, this investigation enrolled 30 patients with facial asymmetry who underwent treatment at our institution between July 2020 and October 2021. We implemented a systematic digital surgical planning protocol and developed specific quantitative parameters to evaluate both the accuracy of digital plan transfer to actual surgery and the degree of symmetry restoration achieved.

## Materials and Methods

### Clinical Materials

A cohort of 30 patients with facial asymmetry deformities (15 males and 15 females) aged 17–30 years (mean age: 22.17 years) underwent treatment at the department of oral and maxillofacial surgery, stomatological hospital, school of stomatology, Southern Medical University from July 2020 to October 2021. Inclusion criteria comprised: (1) pogonion deviation exceeding 4mm from facial midline in natural head position; (2) treatment plan requiring bimaxillary surgery with or without genioplasty; (3) availability of digital orthognathic surgical planning capabilities; and (4) absence of complicating factors including facial trauma, cleft lip and palate, tumors, active temporomandibular joint disorders, hemifacial atrophy, or other related pathologies.

All patients completed prerequisite orthodontic treatment before surgical intervention. Diagnostic records included maxillary and mandibular dental models fabricated by the orthodontics department and cone-beam computed tomography (CBCT) scans acquired using a NewTom device (Italy) in the radiology department. CBCT imaging protocol standardized patient positioning with subjects seated upright, midsagittal plane perpendicular to the floor, Frankfurt horizontal plane parallel to the floor, and dentition in maximum intercuspation. Throughout image acquisition, patients maintained head stability and calm breathing patterns, avoiding deglutition or mastication movements that might compromise image quality.

### Digital Simulation for Surgical Design

CBCT data were imported into Materialise Mimics software (Belgium) in DICOM format for three-dimensional skull reconstruction. Final occlusal relationships were determined using plaster models of the maxillary and mandibular dental arches. These models were digitized using a DS-EX scanner (Xianlin 3D Technology Co., Ltd., China) and imported into Mimics as STL files to replace the CBCT-derived dentition, thereby enhancing accuracy of the digital dental arch representations.

Within the Mimics platform, virtual surgical simulations were performed for LeFort I maxillary osteotomy and bilateral sagittal split ramus osteotomy procedures. The maxilla and mandible were virtually repositioned in three dimensions as integrated units based on the predetermined final occlusal relationship. Upon achieving optimal positioning, surgical guides for maxillary osteotomy and intermediate occlusal splints were designed based on the new maxillary position relative to the unaltered mandibular position. Final occlusal splints were also fabricated according to the planned definitive positions of both jaws. For cases requiring genioplasty, the procedure was simulated by repositioning the chin segment to the desired location, followed by design of custom cutting and repositioning guides.

**Fig. 1:**
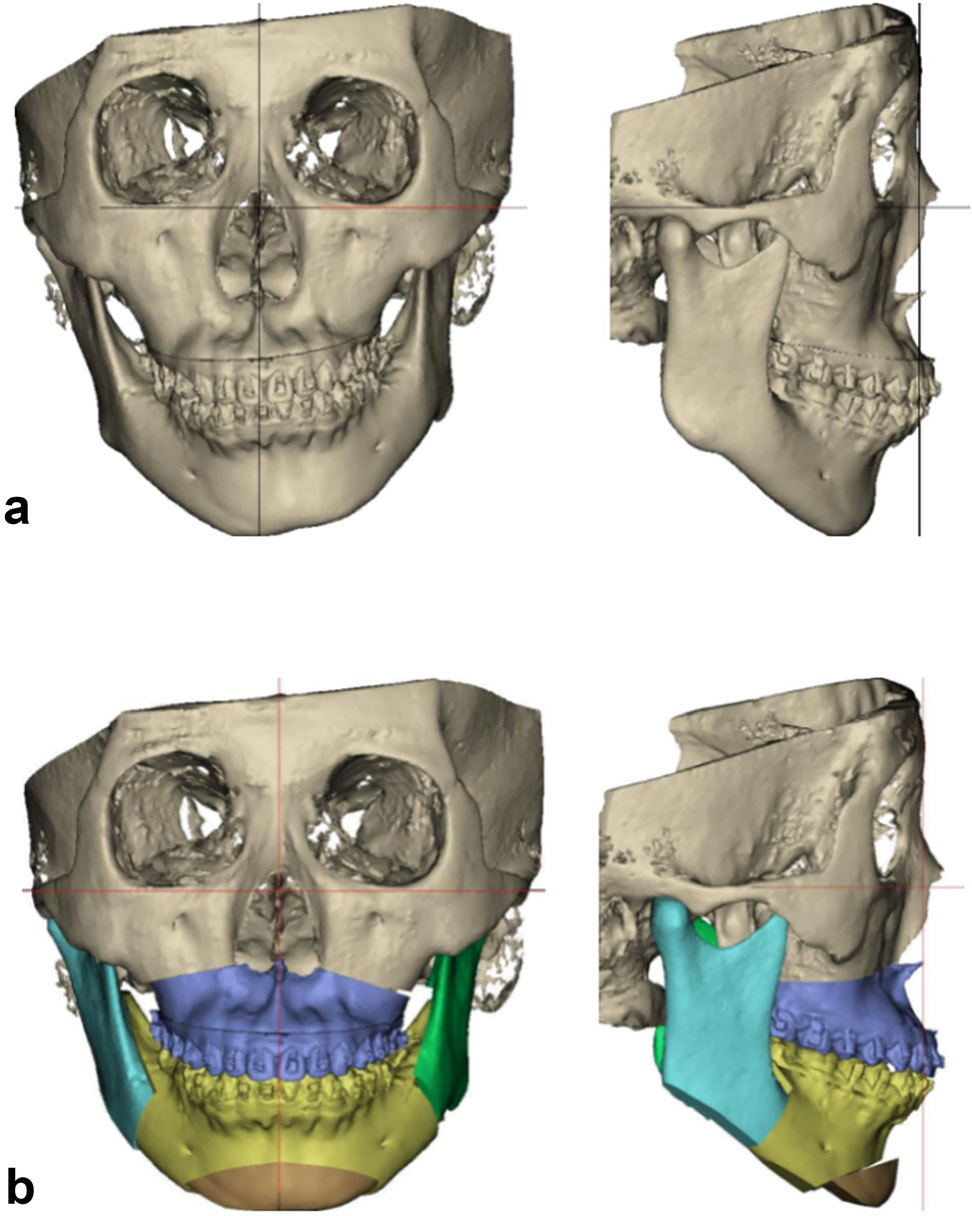
Digitalized simulated surgical design scheme. **a** Preoperative three-dimensional reconstruction of the craniofacial skeleton. **b** Simulated surgical outcome following virtual LeFort I maxillary osteotomy and bilateral sagittal split ramus osteotomy

All surgical guides and splints were exported as STL files and manufactured using a Blackgeek 3D printer (China) with biocompatible photosensitive resin material. This digital workflow was successfully implemented for all 30 patients with technical support from Aibei Medical Technology (Shenzhen) Co., Ltd. and Beijing Digital Healthcare 3D Printing Engineering Research Center (Fig. 1).

### Surgical Procedure Implementation

Preoperative verification included trial fitting of both intermediate and final occlusal splints to ensure unimpeded seating and accurate interdigitation with the dental arches. All 30 patients underwent LeFort I maxillary osteotomy and bilateral sagittal split ramus osteotomy, with 13 patients additionally receiving genioplasty procedures. Surgical anesthesia consisted of combined intravenous-inhalational general anesthesia, supplemented by local infiltration with vasoconstrictor-containing anesthetic solution in the maxillary and mandibular vestibular surgical sites.

The surgical sequence began with LeFort I maxillary osteotomy. Following mucoperiosteal flap elevation, the anterior and lateral maxillary sinus walls were exposed. Custom-designed cutting guides facilitated precise osteotomy placement according to the digital surgical plan. Complete mobilization of the maxilla was achieved through downfracture and removal of bony interferences. The maxilla was then positioned using the intermediate occlusal splint to establish the planned relationship with the unaltered mandible, followed by rigid internal fixation with miniplates and screws.

**Fig. 2:**
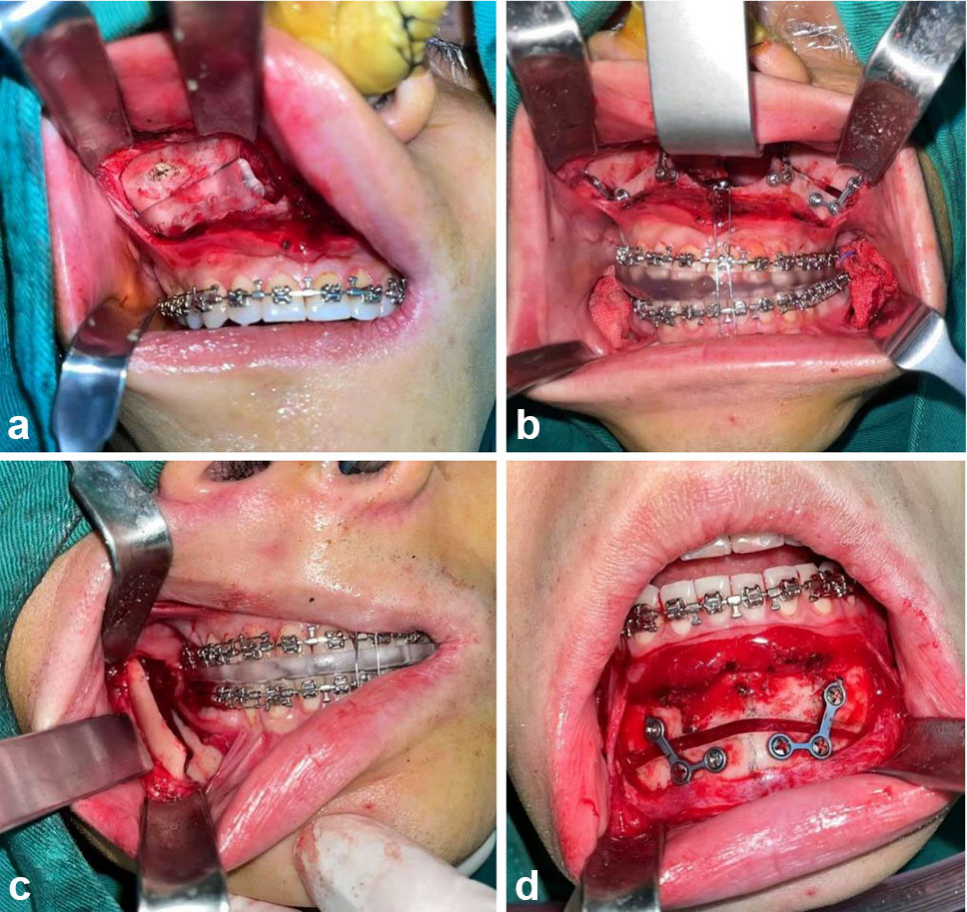
Surgical procedure implementation. **a** Intraoperative marking of the LeFort I maxillary osteotomy line guided by the digitally designed surgical template. **b** Maxillary repositioning and rigid internal fixation with miniplates and screws following accurate placement using the intermediate occlusal splint. **c** Mandibular repositioning and fixation of the bilateral sagittal split ramus osteotomy using the final occlusal splint to achieve planned occlusion. **d** Genioplasty procedure with precise osteotomy and repositioning of the chin segment using the digitally fabricated surgical guide

Bilateral sagittal split ramus osteotomy of the mandible was subsequently performed. After mucoperiosteal flap elevation, standardized horizontal, vertical, and sagittal osteotomies were executed, enabling controlled splitting of the mandibular ramus. The final occlusal splint guided positioning of the tooth-bearing mandibular segment according to the preoperative plan, allowing for appropriate advancement, retraction, or rotation before rigid internal fixation. For the 13 patients requiring genioplasty, the procedure was performed using custom osteotomy guides to execute precise chin segment cuts. The mobilized segment was then repositioned according to the preoperative design and secured with titanium plates and screws in the planned position (Fig. 2).

**Fig. 3:**
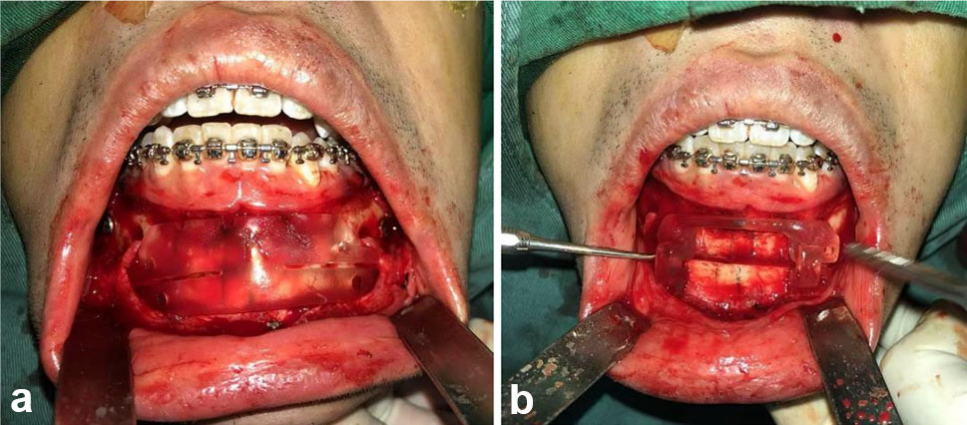
Application of digital technology in genioplasty. **a** Genioplasty osteotomy guide plate positioned on the anterior mandible to establish precise cutting line according to the digital surgical plan. **b** Genioplasty repositioning guide utilized for accurate positioning of the chin segment to the preoperatively designed position before rigid fixation

Figure 3 demonstrates the application of digital technology in genioplasty surgery through customized surgical guides. The genioplasty osteotomy guide plate (Fig. 3a) was precisely positioned on the anterior surface of the mandible to mark the exact cutting line as planned in the preoperative digital simulation. Following osteotomy, the genioplasty repositioning guide (Fig. 3b) was utilized to accurately reposition the chin segment according to the preoperative design. These digitally designed and 3D-printed guides ensured precise implementation of the planned chin correction, contributing to the overall facial symmetry improvement observed in patients undergoing genioplasty.

### The Assessment of Surgical Outcomes

#### Establishment of Reference Planes

**Fig. 4:**
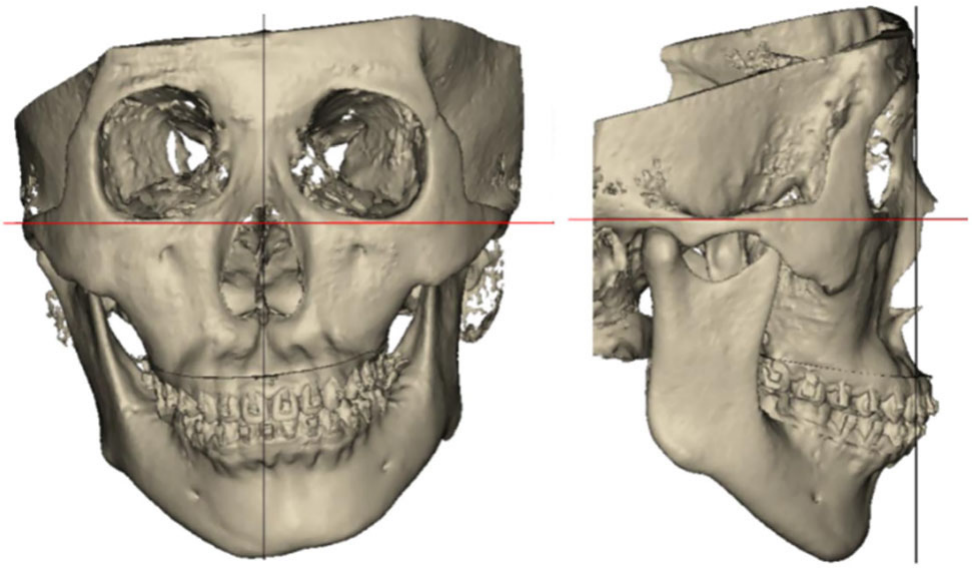
Three reference planes used for quantitative assessment of facial symmetry. The midsagittal plane (SP) connects the bony nasion and sella turcica, perpendicular to the frankfort horizontal plane; the frankfort horizontal plane (FH) passes through the highest points of both external auditory canals and the lowest point of the infraorbital rim; and the coronal plane (CP) passes through the bony nasion perpendicular to both SP and FH planes

Three standardized reference planes were established for quantitative assessment (Fig. 4). The Frankfurt horizontal plane (FH) was defined by connecting the most superior aspects of both external auditory canals and the most inferior point of the infraorbital rim. The midsagittal plane (SP) was constructed through the bony nasion and sella turcica, oriented perpendicular to the Frankfurt horizontal plane. The coronal plane (CP) was established passing through the bony nasion, maintaining perpendicularity to both the Frankfurt horizontal and midsagittal planes. These reference planes provided a consistent three-dimensional coordinate system for all measurements and comparative analyses.

#### Measurement Parameters

**Fig. 5:**
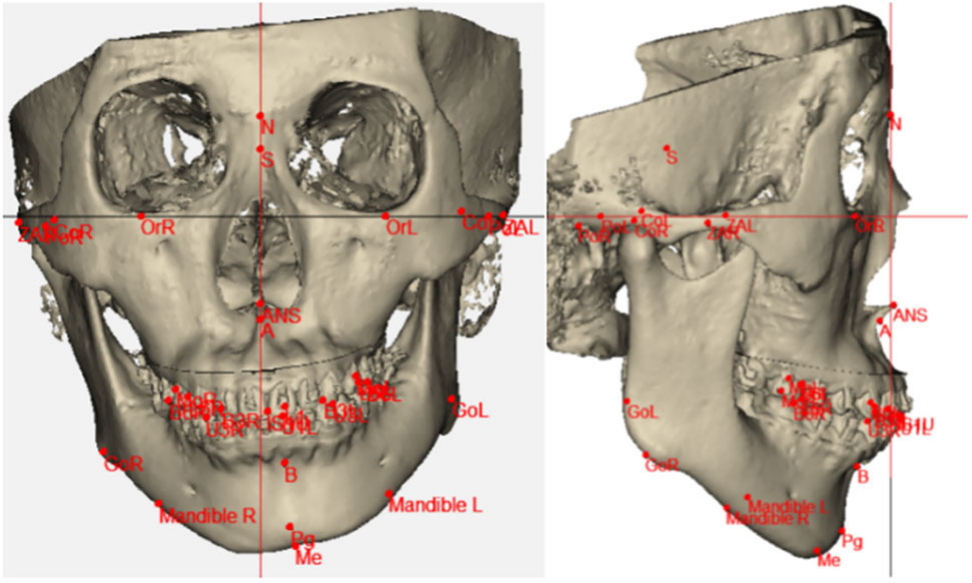
Landmarks for measurement showing the bilateral maxillary and mandibular canine cusps and mesiobuccal cusps of the first molars used for quantitative assessment of surgical outcomes

A comprehensive set of measurement parameters was established to quantify symmetry and surgical accuracy (Fig. 5). These parameters consisted of perpendicular distances from specific dental landmarks to the three reference planes:

Maxillary Parameters: B3L-CP refers to the distance from the cusp of maxillary left canine to the CP. B3L-FH refers to the distance from the cusp of maxillary left canine to the FH. B3L-SP refers to the distance from the cusp of maxillary left canine to the SP. B3R-CP refers to the distance from the cusp of maxillary right canine to the CP. B3R-FH refers to the distance from the cusp of maxillary right canine to the FH. B3R-SP refers to the distance from the cusp of maxillary right canine to the SP. B6L-CP refers to the distance from the mesiobuccal cusp of maxillary left first molar to the CP. B6L-FH refers to the distance from the mesiobuccal cusp of maxillary left first molar to the FH. B6L-SP refers to the distance from the mesiobuccal cusp of maxillary left first molar to the SP. B6R-CP refers to the distance from the mesiobuccal cusp of maxillary right first molar to the CP. B6R-FH refers to the distance from the mesiobuccal cusp of maxillary right first molar to the FH. B6R-SP refers to the distance from the mesiobuccal cusp of maxillary right first molar to the SP.

Mandibular Parameters: U3L-CP refers to the distance from the cusp of mandibular left canine to the CP. U3L-FH refers to the distance from the cusp of mandibular left canine to the FH. U3L-SP refers to the distance from the cusp of mandibular left canine to the SP. U3R-CP refers to the distance from the cusp of mandibular right canine to the CP. U3R-FH refers to the distance from the cusp of mandibular right canine to the FH. U3R-SP refers to the distance from the cusp of mandibular right canine to the SP. U6L-CP refers to the distance from the mesiobuccal cusp of mandibular left first molar to the CP. U6L-FH refers to the distance from the mesiobuccal cusp of mandibular left first molar to the FH. U6L-SP refers to the distance from the mesiobuccal cusp of mandibular left first molar to the SP. U6R-CP refers to the distance from the mesiobuccal cusp of mandibular right first molar to the CP. U6R-FH refers to the distance from the mesiobuccal cusp of mandibular right first molar to the FH. U6R-SP refers to the distance from the mesiobuccal cusp of mandibular right first molar to the SP.

Chin Position Parameter: P-SP refers to the distance from the pogonion to SP (Fig. 5).

#### Evaluation Criteria

Postoperative assessment utilized CBCT data acquired within one week after surgery. Three-dimensional skull reconstructions were generated, and measurements were performed to determine the differences in distances between bilateral landmarks (canine cusps and first molar mesiobuccal cusps) to each reference plane (B3L-FH-B3R, B3L-SP-B3R, B3L-CP-B3R, U3L-FH-U3R, U3L-SP-U3R, U3L-CP-U3R, B6L-FH-B6R, B6L-SP-B6R, B6L-CP-B6R, L6L-FH-L6R, L6L-SP-L6R, L6L-CP-L6R). These measurements facilitated verification of three-dimensional consistency between the actual surgical outcome and preoperative simulation.

Facial symmetry was considered achieved when the maximum difference between corresponding bilateral landmarks relative to all three reference planes was ≤ 1 mm. Additionally, Kondo's formula [8] was applied to calculate asymmetry rates, expressed as (G-K)/G × 100%, where G represents the larger and K the smaller value of distances between corresponding bilateral landmarks and each reference plane. Successful symmetry correction required asymmetry rates < 10% for all measurements. Based on Haraguchi's definition [9] that chin deviation ≥ 2 mm constitutes facial asymmetry, we established a criterion whereby postoperative distance from pogonion to the midsagittal plane (P-SP) must be <2 mm to satisfy symmetry requirements. These comprehensive evaluation criteria enabled objective assessment of both surgical accuracy and treatment efficacy in restoring facial symmetry.

### Statistical Methods

Statistical analysis was performed using SPSS software (version 22.0). All quantitative data were expressed as mean ± standard deviation (SD). Comparative analysis between preoperative simulated surgical design and actual postoperative measurements was conducted using paired sample t-tests. Statistical significance was established at *P *< 0.05 for all analyses.

## Results

### Patient's Surgical Outcomes

All thirty patients with facial asymmetry deformities successfully underwent LeFort I maxillary osteotomy and bilateral sagittal split ramus osteotomy (BSSRO) of the mandible, with thirteen patients additionally receiving simultaneous genioplasty procedures. The surgical interventions were completed without significant complications, with no instances of unexpected fractures, nerve damage, or excessive intraoperative hemorrhage reported in any case.

### Consistency Between Actual Surgery and Preoperative Simulation

Comparative analysis between preoperative simulation and postoperative outcomes was performed using Mimics software. Measurements quantified the distances between bilateral maxillary and mandibular canine cusps and first molar mesiobuccal cusps relative to the three established reference planes (Frankfurt horizontal, midsagittal, and coronal) in both the preoperative simulated models and one-week postoperative three-dimensional reconstructions. Statistical analysis revealed no significant differences between preoperative and postoperative measurements for any of the evaluated parameters (Table 1). These findings demonstrate high fidelity between digital surgical planning and actual surgical execution, confirming that the preoperative virtual designs were accurately transferred to clinical implementation.

**Table 1:** Comparison of measurement indicators between preoperative simulated surgical design and actual surgery

Measurement indicators (mm)	Simulated surgical design (Mean ± SD)	Actual surgery (Mean ± SD)	T-value	*P* value
B3L-FH-B3R	0.28 ± 0.61	0.25 ± 0.85	0.0394	0.9688
B3L-SP-B3R	− 0.08 ± 1.92	− 0.52 ± 2.33	1.690	0.1017
B3L-CP-B3R	0.22 ± 0.78	0.07 ± 1.15	0.9555	0.3472
B6L-FH-B6R	0.74 ± 1.00	0.83 ± 1.01	0.4627	0.6470
B6L-SP-B6R	0.50 ± 1.62	0.39 ± 1.84	0.5091	0.6145
B6L-CP-B6R	− 0.39 ± 1.97	− 0.60 ± 2.36	1.540	0.1345
U3L-FH-U3R	0.16 ± 0.90	0.18 ± 1.02	0.2654	0.7926
U3L-SP-U3R	0.60 ± 1.62	0.26 ± 2.32	1.223	0.2314
U3L-CP-U3R	0.19 ± 1.24	0.46 ± 1.45	1.742	0.0921
L6L-FH-L6R	0.57 ± 1.10	0.63 ± 1.07	0.3196	0.7536
L6L-SP-L6R	0.15 ± 1.94	− 0.59 ± 2.10	1.933	0.0631
L6L-CP-L6R	0.31 ± 1.84	0.27 ± 2.00	0.2991	0.7670

The table presents the differences in distances (mm) between bilateral corresponding landmarks relative to the three reference planes (FH: Frankfort Horizontal plane, SP: midsagittal plane, CP: coronal plane). Values are presented as mean ± standard deviation (SD). Statistical comparison between simulated and actual surgery outcomes was performed using paired t-test, with *P *< 0.05 considered statistically significant.

### Improvement of Facial Symmetry Using Digital-Assisted Techniques

Postoperative measurements were conducted on all 30 patients to evaluate symmetry outcomes. Analysis of the difference in distances between bilateral maxillary and mandibular canine cusps and first molar mesiobuccal cusps relative to the three reference planes revealed that all measurements remained within the established criterion of ≤1mm difference between corresponding landmarks (Table 1). This indicates successful achievement of the primary symmetry objective according to our established parameters.

Application of Kondo's asymmetry rate formula revealed varying degrees of symmetry restoration across different landmarks and planes. The postoperative asymmetry rates for bilateral maxillary canine cusps relative to the SP, FH, and CP were 13.12 ± 10.05%, 1.40 ± 0.99%, and 29.08 ± 22.81%, respectively. Bilateral maxillary first molar mesiobuccal cusps demonstrated asymmetry rates of 6.00 ± 4.89%, 2.24 ± 1.53%, and 7.48 ± 7.15% relative to the SP, FH, and CP, respectively. For the mandibular dentition, asymmetry rates for bilateral canine cusps were 9.40 ± 7.76%, 1.68 ± 1.10%, and 28.89 ± 25.46% relative to the SP, FH, and CP, respectively, while mandibular first molar mesiobuccal cusps showed rates of 6.72 ± 5.36%, 2.03 ± 1.40%, and 7.73 ± 6.73% relative to the SP, FH, and CP, respectively. These findings indicate that 75% of all measured parameters achieved the target asymmetry rate of <10%.

**Fig. 6:**
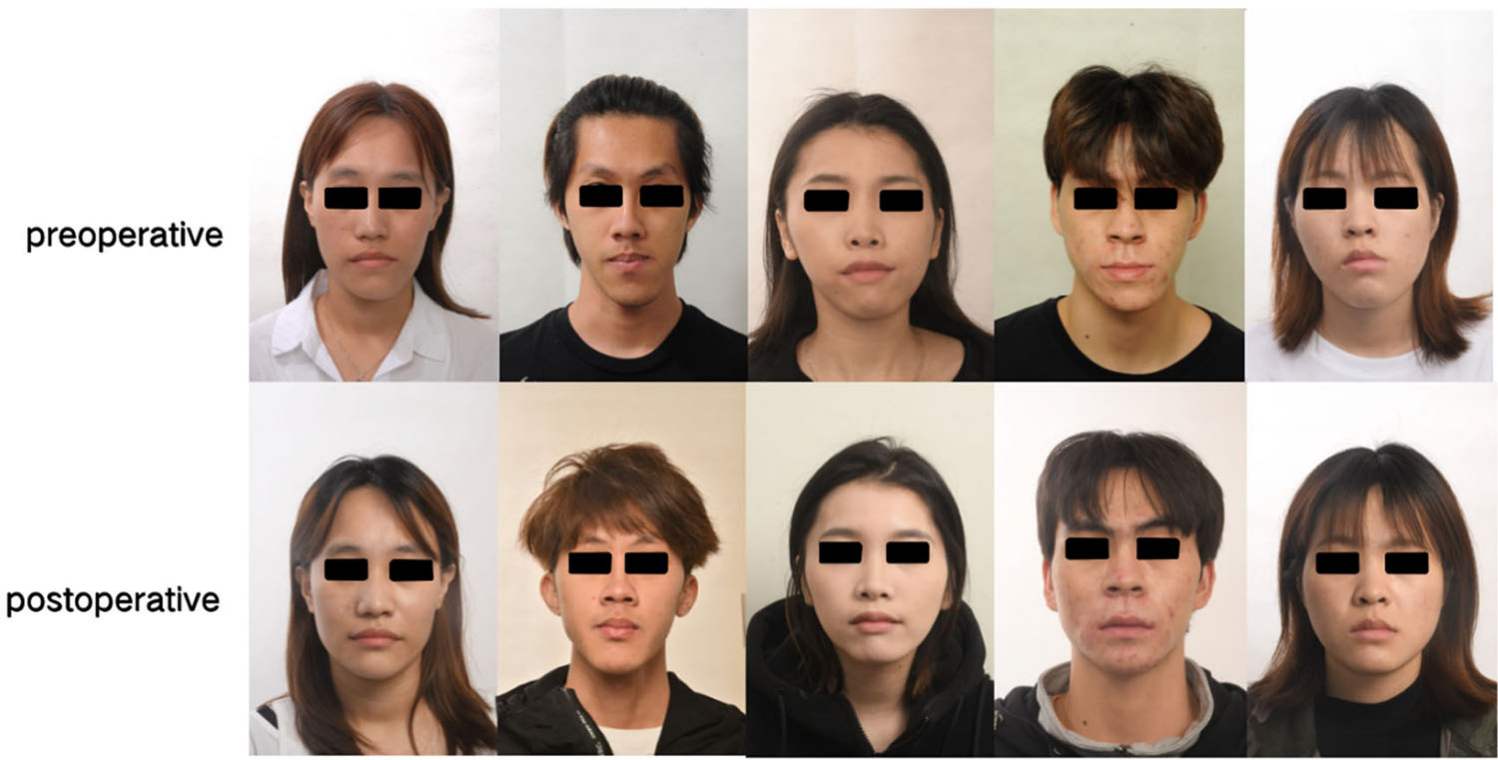
The comparison of preoperative and postoperative frontal views in patients with facial asymmetry

Assessment of chin position revealed an average distance from pogonion to the midsagittal plane of 1.82 ± 1.16mm across all postoperative patients, falling within our established criterion of < 2 mm for successful chin symmetry correction. Clinical evaluation of the 30 patients further demonstrated significant improvement in overall facial symmetry and establishment of favorable occlusal relationships, resulting in high patient satisfaction with postoperative facial appearance (Fig. 6).

**Fig. 7:**
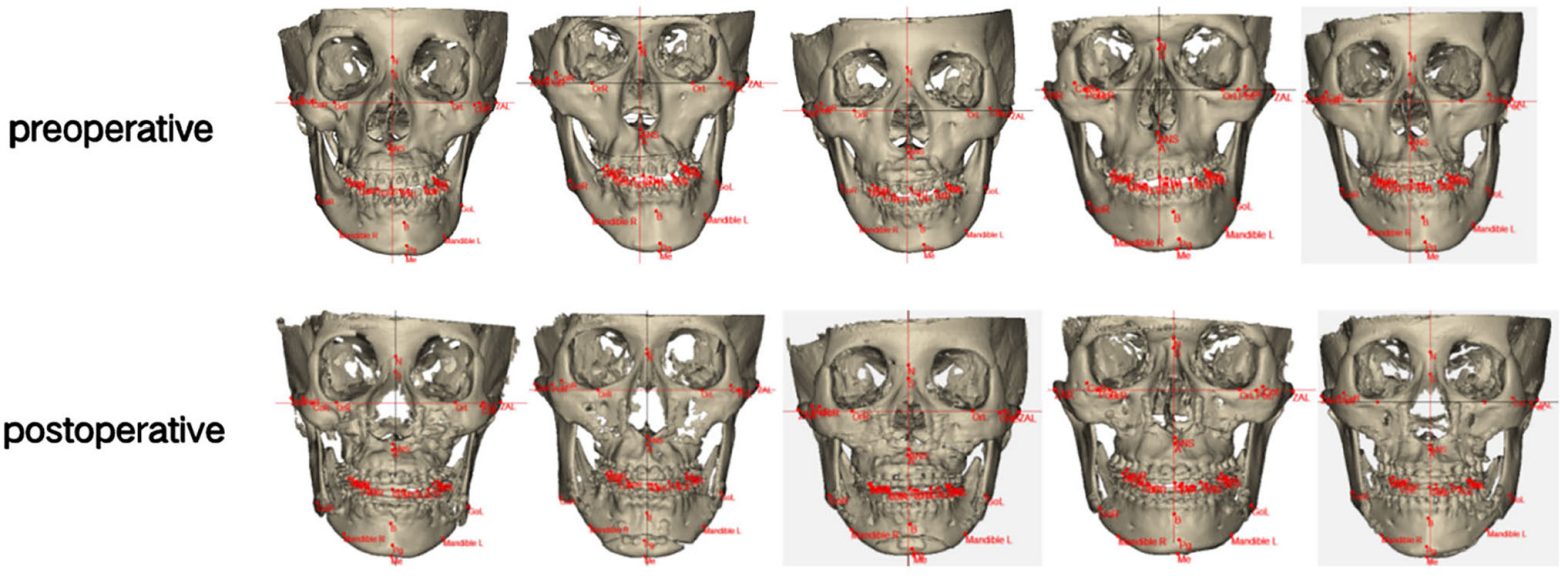
Comparison of preoperative (upper row) and postoperative (lower row) 3D cranial reconstructions in five representative patients with facial asymmetry

A visual comparison of preoperative and postoperative 3D cranial reconstructions further demonstrates the significant improvement in facial symmetry (Fig. 7). The 3D models clearly show the correction of chin deviation and overall facial symmetry achieved through digital technology-assisted orthognathic surgery. These visual results correlate with our quantitative measurements and highlight the effectiveness of our surgical approach in correcting facial asymmetry.

## Discussion

This study used digital technology for surgical simulation in 30 facial asymmetry patients, creating customized guides for surgery. All patients achieved favorable outcomes with satisfactory occlusion and facial appearance. No statistically significant differences were found between planned and actual surgical measurements, confirming accurate transfer of digital designs to clinical implementation. Postoperative measurements showed excellent symmetry with bilateral landmark discrepancies ≤ 1 mm, pogonion deviation < 2 mm, and 75% of asymmetry rates below 10%. Only measurements between maxillary canine cusps and certain reference planes exceeded the 10% threshold. These results demonstrate good facial symmetry outcomes and validate the effectiveness of digital technology in orthognathic surgery for facial asymmetry.

For the cases where the asymmetry rate of the distance difference between bilateral maxillary canine cusps and the SP is slightly greater than 10% in research results, there are two possible reasons. One is related to insufficient symmetry correction of dental arch during preoperative orthodontic treatment in some patients. The other possibility is related to inconsistent judgment of alignment between maxillary dental midline and facial midline caused by the impact of general anesthesia through nasal intubation, leading to inconsistent positioning of maxillary dental midline with facial midline in some patients. Therefore, we need to strengthen the correction of preoperative dental arch symmetry and accurately assess the alignment between the midline of maxillary dental arch and the facial midline during surgery. In cases where the asymmetry rate of the distance difference between bilateral maxillary canine cusps and CP is greater than 10%, the relationship between facial asymmetry and soft/hard tissue asymmetry appears to be a contributing factor in some patients. In these cases, bone tissue requires excessive rotation in the horizontal plane to compensate for insufficient soft tissue, ultimately resulting in a significant difference in distance between bilateral maxillary canine cusps and CP.

While three-dimensional CT technology has been widely applied in orthognathic surgery planning, our study makes distinct contributions to the existing literature through several innovations. Although digital technology-assisted design has been reported in various studies, each publication focuses on different types of dentofacial deformities, employs different reference planes, measurement indicators, and evaluation standards. Furthermore, each medical institution and surgeon may have their own unique approaches and philosophies regarding digital-assisted surgical design. Our study specifically focuses on facial asymmetry deformities—among the most challenging to achieve satisfactory surgical outcomes—using consistent, reproducible reference planes, measurement indicators, and evaluation standards that reflect our institution's surgical philosophy. We established a comprehensive quantitative assessment framework using three reference planes (midsagittal, frankfort horizontal, and coronal planes) with specific thresholds (≤ 1 mm landmark discrepancies and < 10% asymmetry rates) to evaluate facial symmetry, providing more precise metrics than typical qualitative assessments. Our analysis incorporated multiple anatomical landmarks (canine cusps and first molar mesiobuccal cusps) rather than focusing solely on the pogonion, offering more comprehensive hard tissue symmetry data. Additionally, we quantitatively measured the accuracy of transferring digital plans to actual surgery, validating the precision of the digital workflow. Unlike studies reporting only successful outcomes, we thoroughly analyzed cases not achieving ideal symmetry, identifying contributing factors and suggesting clinical improvements. The research revealed significant correlations between preoperative orthodontic preparation and postoperative outcomes, providing important evidence for multidisciplinary collaboration. We also identified the previously under-reported impact of nasal intubation anesthesia on midline judgment during surgery. Furthermore, our exploration of compensatory rotation mechanisms of bone tissue in soft-hard tissue asymmetry scenarios offers new insights into facial symmetry correction biomechanics. The detailed documentation of our complete digital workflow from CBCT acquisition to surgical guide fabrication provides a reproducible protocol for other centers. These contributions collectively advance assessment methodology, clinical problem-solving, and multidisciplinary collaboration in the challenging arena of facial asymmetry correction, extending well beyond merely validating digital technology.

The clinical significance of digital technology in orthognathic surgery for facial asymmetry extends beyond technical advantages to substantive improvements in patient care. Three-dimensional planning enables surgeons to perform detailed analyses of facial asymmetry, accurately plan complex movements, and predict soft tissue changes more precisely than traditional 2D methods [10]. This technology fundamentally transforms the diagnostic process by allowing for comprehensive visualization of skeletal discrepancies from multiple angles, revealing asymmetries that might be overlooked in conventional approaches. The integration of various imaging modalities through digital platforms provides a holistic evaluation of skeletal, dental, and soft tissue disharmonies [11], enabling clinicians to develop treatment plans that address all aspects of facial asymmetry. Digital simulation of different surgical scenarios helps determine the most effective interventions tailored to individual patient needs [4], enhancing the decision-making process and improving communication between surgical team members. From a surgical execution perspective, digital technology supports the creation of precise surgical guides and splints that translate virtual plans into actual surgical procedures with high fidelity, significantly reducing intraoperative guesswork and improvisation. This improved surgical precision has demonstrated better alignment of dental midlines and mandibular planes compared to traditional methods [12], contributing to more balanced facial aesthetics. The combination of digital planning with contemporary surgical approaches has not only improved precision but also shortened treatment time while enhancing aesthetic outcomes [13], addressing key patient concerns. Additionally, digital technology offers substantial benefits for patient education and informed consent, as three-dimensional visualizations of anticipated surgical outcomes help patients develop realistic expectations and increase their involvement in treatment decisions. The integration of 3D printing and virtual surgical planning has further improved surgical predictability [14], while studies confirm that fully digital approaches effectively treat complex skeletal malocclusions with clinically acceptable results [15]. Collectively, these technological advancements contribute to improved patient satisfaction and quality of life post-surgery [16], ultimately fulfilling the primary clinical goal of orthognathic surgery for facial asymmetry.

Digital technology demonstrates significant advantages over conventional methods in orthognathic surgery for facial asymmetry. While traditional approaches rely heavily on the surgeon's manual skills and experience, digital planning offers superior precision with studies showing mean system errors of only 0.94 mm for orthognathic surgery [17]. Quantitative assessments revealed that virtual surgical planning (VSP) results in significantly greater improvements in facial midline and overall facial symmetry compared to conventional approaches, with studies demonstrating a mean improvement in facial symmetry scores [18, 19]. Additionally, VSP yields better accuracy in jaw repositioning, with statistically significant improvements in cephalometric measurements compared to traditional planning [20]. Digital planning enables precise three-dimensional positioning of jaw segments, resulting in better alignment of the lower interincisal point, mandibular sagittal plane, and centering of dental midlines [12, 21]. Furthermore, digital methods eliminate the need for physical articulators and plaster models, reducing laboratory work time and increasing efficiency [22]. VSP also significantly reduces operation duration and improves splint accuracy [23]. However, it's worth noting that despite the quantitative advantages of digital methods, patient satisfaction and quality of life improvements are high for both digital and conventional approaches, suggesting that the subjective experience may not differ significantly between the two methods [18, 24].

Despite the clear clinical benefits of digital technology in orthognathic surgery for facial asymmetry, several practical considerations merit attention. Regarding cost-effectiveness, digital planning methods eliminate the need for physical articulators and plaster models, reducing material costs and laboratory work time [22]. While initial investments in hardware, software, and training are substantial, the long-term savings from improved surgical efficiency and reduced revision surgeries can offset these costs [25]. In terms of accessibility, digital platforms for planning orthognathic surgery are becoming more widely available, allowing for broader implementation of advanced surgical planning techniques [4]. Open-source software and widely available 3D imaging technologies can democratize access to digital planning, making it feasible for smaller practices, though availability still varies by region [22, 25]. The learning curve presents perhaps the most significant challenge, requiring practitioners to acquire new skills in software use and 3D modeling [26]. Our experience aligns with recent studies showing significant annual improvements in surgical accuracy from 2016 to 2020, supporting the presence of a learning curve in digital planning implementation [27]. Extra care is particularly needed in cases involving posterior impaction or greater magnitude of jaw movements to achieve satisfactory surgical accuracy [27]. Nevertheless, the improved precision and patient outcomes provide strong incentives for practitioners to overcome these hurdles, and collaborative approaches between surgeons, technologists, and educators can facilitate the integration of digital planning into routine clinical practice [24].

## Conclusion

Digital technology provides significant advantages over conventional methods in orthognathic surgery for facial asymmetry deformities. While traditional approaches using two-dimensional cephalometric measurements and plaster models cannot intuitively simulate complex three-dimensional movements, digital technology offers comprehensive visualization and precise surgical planning. The application of digital techniques allows for accurate simulation of bone movement, better prediction of surgical outcomes, and fabrication of customized surgical guides that effectively transfer virtual plans to the operating room. Our study demonstrates that this approach results in marked improvement in facial symmetry and enhanced patient satisfaction. Specifically, our quantitative analysis of 30 patients revealed no statistically significant differences between planned and actual surgical outcomes, with bilateral landmark discrepancies consistently ≤ 1 mm, 75% of asymmetry rates below 10%, and mean pogonion deviation of only 1.82 ± 1.16 mm from the midsagittal plane. These measurable outcomes confirm the precision and efficacy of digital surgical planning in facial asymmetry correction. Furthermore, digital technology enables systematic postoperative evaluation, facilitating quality assessment and continuous improvement in surgical techniques. As digital technology continues to evolve in clinical practice, treatment for facial asymmetry is advancing from standardized approaches to more personalized and predictable solutions.

## Data Availability

The datasets used and/or analysed during the current study available from the corresponding author on reasonable request.

## References

[1] Ko EW, Huang CS, Chen YR. Characteristics and corrective outcome of face asymmetry by orthognathic surgery. J Oral Maxillofac Surg. 2009;67:2201–9. 19761914 10.1016/j.joms.2009.04.039

[2] Wang TT, Wessels L, Hussain G, Merten S. Discriminative thresholds in facial asymmetry: a review of the literature. Aesthet Surg J. 2017;37:375–85. 28200081 10.1093/asj/sjw271

[3] Lin HH, Chang HW, Wang CH, Kim SG, Lo LJ. Three-dimensional computer-assisted orthognathic surgery: experience of 37 patients. Ann Plast Surg. 2015;74(Suppl 2):S118-126. 25785379 10.1097/SAP.0000000000000455

[4] Cintra O, Grybauskas S, Vogel CJ, Latkauskiene D, Gama NA Jr. Digital platform for planning facial asymmetry orthodontic-surgical treatment preparation. Dental Press J Orthod. 2018;23:80–93. 30088569 10.1590/2177-6709.23.3.080-093.sar PMC6072444

[5] Zinser MJ, Mischkowski RA, Sailer HF, Zöller JE. Computer-assisted orthognathic surgery: feasibility study using multiple CAD/CAM surgical splints. Oral Surg Oral Med Oral Pathol Oral Radiol. 2012;113:673–87. 22668627 10.1016/j.oooo.2011.11.009

[6] Heufelder M, Wilde F, Pietzka S, Mascha F, Winter K, Schramm A, Rana M. Clinical accuracy of waferless maxillary positioning using customized surgical guides and patient specific osteosynthesis in bimaxillary orthognathic surgery. J Craniomaxillofac Surg. 2017;45:1578–85. 28793965 10.1016/j.jcms.2017.06.027

[7] Bai S, Shang H, Liu Y, Zhao J, Zhao Y. Computer-aided design and computer-aided manufacturing locating guides accompanied with prebent titanium plates in orthognathic surgery. J Oral Maxillofac Surg. 2012;70:2419–26. 22516840 10.1016/j.joms.2011.12.017

[8] Kondo E. Posteroanterior cephalometric study of cranio-facial and arch widths. Nippon Kyosei Shika Gakkai Zasshi. 1972;31:117–36. 4514711

[9] Haraguchi S, Takada K, Yasuda Y. Facial asymmetry in subjects with skeletal Class III deformity. Angle Orthod. 2002;72:28–35. 11843270 10.1043/0003-3219(2002)072<0028:FAISWS>2.0.CO;2

[10] Hsu PJ, Denadai R, Pai BCJ, Lin HH, Lo LJ. Outcome of facial contour asymmetry after conventional two-dimensional versus computer-assisted three-dimensional planning in cleft orthognathic surgery. Sci Rep. 2020;10:2346. 32047228 10.1038/s41598-020-58682-4 PMC7012815

[11] Bennici O, Malgioglio A, Moschitto S, Spagnuolo G, Lucchina AG, Ronsivalle V, Isola G, Giudice AL. A full computerized workflow for planning surgically assisted rapid palatal expansion and orthognathic surgery in a skeletal class III patient. Case Rep Dent. 2022;2022:6413898. 36312572 10.1155/2022/6413898 PMC9605851

[12] De Riu G, Meloni SM, Baj A, Corda A, Soma D, Tullio A. Computer-assisted orthognathic surgery for correction of facial asymmetry: results of a randomised controlled clinical trial. Br J Oral Maxillofac Surg. 2014;52:251–7. 24418178 10.1016/j.bjoms.2013.12.010

[13] Kong L, Liu X, Zhang J. Combining a digital design-mediated surgery-first approach and clear aligners to treat a skeletal Class III defect for aesthetic purposes: a case report. J Int Med Res. 2022;50:3000605221094524. 35485854 10.1177/03000605221094524 PMC9067056

[14] Zoabi A, Redenski I, Oren D, Kasem A, Zigron A, Daoud S, Moskovich L, Kablan F, Srouji S. 3D printing and virtual surgical planning in oral and maxillofacial surgery. J Clin Med. 2022;11:2385. 35566511 10.3390/jcm11092385 PMC9104292

[15] Li M, Shen S, Zhao Z, Wang B, Yu H. The application of a fully digital approach in the treatment of skeletal class III malocclusion: a preliminary study. BMC Oral Health. 2023;23:237. 37095513 10.1186/s12903-023-02918-y PMC10124042

[16] Kim YJ, Kim MY, Jha N, Jung MH, Kwon YD, Shin HG, Ko MJ, Jun SH. Treatment outcome and long-term stability of orthognathic surgery for facial asymmetry: a systematic review and meta-analysis. Korean J Orthod. 2024;54:89–107. 38533597 10.4041/kjod23.194 PMC10973727

[17] Tanikawa C, Yamashiro T. Development of novel artificial intelligence systems to predict facial morphology after orthognathic surgery and orthodontic treatment in Japanese patients. Sci Rep. 2021;11:15853. 34349151 10.1038/s41598-021-95002-w PMC8339122

[18] Liao YF, Chen YA, Chen YC, Chen YR. Outcomes of conventional versus virtual surgical planning of orthognathic surgery using surgery-first approach for class III asymmetry. Clin Oral Investig. 2020;24:1509–16. 32100114 10.1007/s00784-020-03241-4

[19] Lo LJ, Yang CT, Ho CT, Liao CH, Lin HH. Automatic assessment of 3-dimensional facial soft tissue symmetry before and after orthognathic surgery using a machine learning model: a preliminary experience. Ann Plast Surg. 2021;86:S224-s228. 33443885 10.1097/SAP.0000000000002687

[20] Barone M, De Stefani A, Baciliero U, Bruno G, Gracco A. The accuracy of jaws repositioning in bimaxillary orthognathic surgery with traditional surgical planning compared to digital surgical planning in skeletal class III patients: A retrospective observational study. J Clin Med. 2020;9:1840. 32545621 10.3390/jcm9061840 PMC7355953

[21] Choi J. Computer-aided design/computer-aided manufacturing assisted orthognathic surgery. Int J Oral Maxillofac Surg. 2024;52:124–5.

[22] Stamm T, Böttcher D, Kleinheinz J. The University Münster model surgery system for orthognathic surgery—the digital update. Head Face Med. 2021;17:31. 34301272 10.1186/s13005-021-00278-y PMC8299672

[23] Schneider D, Kämmerer PW, Hennig M, Schön G, Thiem DGE, Bschorer R. Customized virtual surgical planning in bimaxillary orthognathic surgery: a prospective randomized trial. Clin Oral Investig. 2019;23:3115–22. 30443778 10.1007/s00784-018-2732-3

[24] Lee YC, Kim SG. Redefining precision and efficiency in orthognathic surgery through virtual surgical planning and 3D printing: a narrative review. Maxillofac Plast Reconstr Surg. 2023;45:42. 38108939 10.1186/s40902-023-00409-2 PMC10728393

[25] Kim J-Y, Lee Y-C, Kim S-G, Garagiola U. Advancements in oral maxillofacial surgery: a comprehensive review on 3D printing and virtual surgical planning. Appl Sci. 2023;13:9907.

[26] Kim Y-J, Gil B-G, Ryu J-J. Application of CAD-CAM technology to surgery-first orthognathic approach. J Korean Dental Assoc. 2018;56:622–30.

[27] Beek D-M, Baan F, Liebregts J, Nienhuijs M, Bergé S, Maal T, Xi T. A learning curve in 3D virtual surgical planned orthognathic surgery. Clin Oral Invest. 2023;27:3907–15. 10.1007/s00784-023-05013-2 PMC10329591 37083986

